# Reduced Neural Integration of Letters and Speech Sounds in Dyslexic Children Scales with Individual Differences in Reading Fluency

**DOI:** 10.1371/journal.pone.0110337

**Published:** 2014-10-16

**Authors:** Gojko Žarić, Gorka Fraga González, Jurgen Tijms, Maurits W. van der Molen, Leo Blomert, Milene Bonte

**Affiliations:** 1 Department of Cognitive Neuroscience, Faculty of Psychology and Neuroscience, University of Maastricht, Maastricht, Netherlands; 2 Maastricht Brain Imaging Center (M-BIC), Maastricht, Netherlands; 3 Department of Psychology, University of Amsterdam, Amsterdam, Netherlands; 4 IWAL Institute for Dyslexia, Amsterdam, Netherlands; 5 Rudolf Berlin Center, Amsterdam, Netherlands; 6 Amsterdam Brain and Cognition, University of Amsterdam, Amsterdam, Netherlands; Max Planck Institute for Human Cognitive and Brain Sciences, Germany

## Abstract

The acquisition of letter-speech sound associations is one of the basic requirements for fluent reading acquisition and its failure may contribute to reading difficulties in developmental dyslexia. Here we investigated event-related potential (ERP) measures of letter-speech sound integration in 9-year-old typical and dyslexic readers and specifically test their relation to individual differences in reading fluency. We employed an audiovisual oddball paradigm in typical readers (n = 20), dysfluent (n = 18) and severely dysfluent (n = 18) dyslexic children. In one auditory and two audiovisual conditions the Dutch spoken vowels/a/and/o/were presented as standard and deviant stimuli. In audiovisual blocks, the letter ‘a’ was presented either simultaneously (AV0), or 200 ms before (AV200) vowel sound onset. Across the three children groups, vowel deviancy in auditory blocks elicited comparable mismatch negativity (MMN) and late negativity (LN) responses. In typical readers, both audiovisual conditions (AV0 and AV200) led to enhanced MMN and LN amplitudes. In both dyslexic groups, the audiovisual LN effects were mildly reduced. Most interestingly, individual differences in reading fluency were correlated with MMN latency in the AV0 condition. A further analysis revealed that this effect was driven by a short-lived MMN effect encompassing only the N1 window in severely dysfluent dyslexics versus a longer MMN effect encompassing both the N1 and P2 windows in the other two groups. Our results confirm and extend previous findings in dyslexic children by demonstrating a deficient pattern of letter-speech sound integration depending on the level of reading dysfluency. These findings underscore the importance of considering individual differences across the entire spectrum of reading skills in addition to group differences between typical and dyslexic readers.

## Introduction

Though many children learn to read without any problems, 5% to 10% of children are affected by developmental dyslexia and never acquire proficient reading skills despite normal cognitive abilities and schooling opportunities [1], [2]. A lack of reading fluency has been pinpointed as the most persistent and impaired characteristic of developmental dyslexia [3], although studies also show a large inter-individual variability in the level of reading (dys)fluency [4], [5]. A first important step in obtaining reading fluency in alphabetic orthographies, the formation of solid letter-speech sound correspondences, poses an immediate hurdle for beginning dyslexic readers [6]–[9]. Accordingly, in addition to tackling well-known difficulties in phonological processing, i.e. recognizing and manipulating the sound structure of language [10], [11], many dyslexia interventions contain a condition focused on teaching letter-speech sound correspondences [12]–[14]. In recent years, the neurofunctional basis of the difficulties in building these correspondences has been elucidated by neuroimaging findings showing reduced neural integration of letters and speech sounds in dyslexic children [15]–[17] and adults [18]. How this reduced neural integration scales with individual differences in the level of reading (dys)fluency remains an interesting and open question.

In largely transparent languages, accurate letter-speech sound identification and discrimination is typically observed after one year of reading instruction [19]. However, evidence from neuroimaging studies shows a dissociation between knowing which letters belong to which speech sounds and the automatic neural integration of these associations, with the latter showing a much more protracted period of incremental learning throughout primary school in typical readers [20], [21]. One of the first studies in this domain recorded magnetoencephalography (MEG) in literate Finnish adults and indicated a dynamic process of letter-speech sound integration with audiovisual convergence starting around 225 ms in bilateral temporal cortices [22]. Subsequent functional magnetic resonance imaging (fMRI) evidence revealed the involvement of the superior temporal sulcus, as well as auditory cortex (superior temporal gyrus/planum temporale) [23]. More specifically, results indicated a modulating influence of letters on speech sounds as a consequence of feedback from the superior temporal sulcus to the superior temporal gyrus, but only if the letter and speech sound were presented simultaneously and in a passive listening paradigm [23]–[25].

Evidence for the protracted developmental time-course of letter-speech sound integration comes from a series of studies measuring electroencephalography (EEG) responses in a passive crossmodal “oddball” paradigm [16], [21], [26]. In an oddball paradigm, at a latency of 100–200 ms, a mismatch negativity (MMN) response is elicited by a rare sound (deviant, or oddball) that is presented in a sequence of frequent (standard) sounds [27]. The MMN represents an automatic change detection response sensitive to deviation from traces in auditory short-term memory [27], [28]. Multiple experiments have shown that the MMN is sensitive to language-specific speech sounds in adults and in children [27], [29]–[37], as well as to audiovisual integration [21], [26], [38]–[42]. Furthermore, in school-aged children the speech evoked MMN response may be followed by an additional late negativity (Late MMN or LN) in a broader time window from 300 to 700 ms, the amplitude of which decreases in adulthood [43]–[45], suggesting fast and automatic discrimination and representation of speech syllables in adults while the developing brain still needs additional processing resources to reach to the same result. In the paradigm of Froyen and colleagues [16], [21], [26], in an auditory and two audiovisual experimental blocks, the Dutch speech sound/a/was presented as the standard stimulus, and the Dutch speech sound/o/as the deviant stimulus. In the audiovisual blocks, the letter ‘a’ was presented together with the standard and deviant speech sounds, leading to a double violation: the deviant speech sound/o/violated the expectation built by both the standard speech sound/a/and the letter ‘a’. In order to investigate the temporal window of integration the letter ‘a’ was presented either simultaneously, or 200 ms before speech sound onset. Due to the double violation, literate adults showed a significant enhancement of the MMN response in the audiovisual as compared to auditory blocks, but, reminiscent of the fMRI findings [23]–[25], only when letters and speech sounds were presented simultaneously, indicating an early and automatic audiovisual integration [26]. Using the same paradigm, 8 year-old typically reading children after one year of reading instruction only showed a significant audiovisual enhancement of a late negativity (LN) evoked around 650 ms after speech sound onset and only when the letter preceded the speech sound, indicating a slow and immature neural integration of letters and speech sounds [21]. A more developed, bit still not adult-like pattern was apparent in 11 year-old typically reading children after four years of reading instruction. That is, these more advanced readers showed a cross-modal enhancement of the earlier MMN when the letter preceded the speech sound, and a cross-modal LN enhancement when the letter and speech sound were presented simultaneously [21]. Although adults did not show these late letter effects in this passive paradigm using simple speech stimuli [26], orthographic influences on spoken language processing around 400–700 ms have been reported in adults when using a more complex metaphonological task [46], [47]. Thus, whereas the observed cross-modal MMN enhancement may be a neurophysiological marker of initial and automatic letter-speech sound integration and/or representation [27], [28]), the crossmodal LN enhancement may reflect more elaborate, explicit associative processes that, in children but not in adults, are recruited for the integration of simple letter-speech pairs.

The same crossmodal MMN paradigm [16], [21], [26], was used in 11 year-old dyslexic children after 4 years of reading instruction, and indicated reduced integration of letters and speech sounds, with significant letter effects only in the late LN window [16]. Further evidence for a reduced sensitivity to letter-speech sound associations in dyslexia comes from fMRI studies showing an underactivation of superior temporal cortex during letter-speech sound integration in 9-year-old dyslexic children [15] and adults [18], as well as from the absence of a crossmodal MMN enhancement for spoken-written syllable pairs in dyslexic adults [41]. Similarly, recent studies suggest reduced orthographic-phonological integration in dyslexics on word reading tasks [48]–[51].

In the current study, we investigate EEG measures of letter-speech sound integration in 9-year-old typical and dyslexic readers and specifically test whether they scale with behavioral measures of reading fluency and reading-related skills. To this end we employ an adapted version of the audiovisual paradigm of Froyen and colleagues [16] in typical readers (n = 20) and age-matched dyslexic children (n = 36) with variable levels of reading (dys)fluency. By focusing on 9-year old children, we extend previous ERP findings to a different age group, 2.5 years after reading instruction, intermediate between the previously studied age groups, namely typical readers with 1 or 4 years of reading instruction [21] and dyslexic readers with 4 years of reading instruction [16]. This age range also coincides with the age of participants from a previous fMRI study reporting reduced neural letter-speech sound integration in dyslexics, as well as correlations between these neural measures and behavioral reading scores [15], allowing us to further investigate the same processes with a different technique at the same level of development. As the morphology, amplitude and timing of ERP components are known to be age dependent [52]–[56], we chose to use a chronologically age matched control group, rather than a reading age matched control group. Finally, including a larger group of dyslexic children with variable levels of reading fluency, allowed us to demonstrate brain-behavior relations not only across typical and dyslexic readers but also to show interindividual differences within the group of dyslexic readers.

## Methods

### Participants

In total, 61 children (41 dyslexic and 20 typical readers) participated in the EEG experiment. All children were 3^rd^ graders, native Dutch speakers, having received 2.5 years of reading instruction. Data of 56 children were included in the analysis, including 36 dyslexic readers (9.0 years old, range: 8.2–9.9; 16 girls; 6 left-handed, as assessed by an adapted version of Annett’s handedness questionnaire [57], and 20 age-matched typical readers (8.8 years old range: 8.3–9.5; 12 girls; 2 left-handed). Data of five children with dyslexia were discarded, 4 children did not complete the EEG measurement and 1 child moved excessively during the EEG measurement.

Dyslexic children were recruited from a specialized institute for dyslexia and reading problems. Prior to the present study, they were diagnosed as dyslexic after an extensive cognitive psycho-diagnostic procedure by the institute. Each of the dyslexic children scored in the lowest 10 percentile of the age appropriate group on standard reading tests (see below). Other behavioral scores such as phonological skills or rapid automatized naming (RAN), were not included as selection criteria. Typical readers had reading score percentile of 25 or higher and were recruited from 3^rd^ grades of primary schools in the same area and had similar social-demographic characteristics. All children had a normal hearing and normal or corrected to normal vision, as reported by parents. Comorbidity with behavioral and/or attention disorders was assessed with the Child Behavior Checklist (CBCL) from the Achenbach system of empirically based assessment (ASEBA) [58] which was completed by the parents, and used as an exclusion criterion. Children with below average IQ were also excluded from the experiment. Written informed consent was obtained from the parents of each child. Each child received a small present after the experiment and travel costs were reimbursed. The approval for the research was obtained from a local ethical committee of the Developmental Psychology department of the University of Amsterdam.

### Behavioral tests

Prior to the EEG measurements, each of the children performed standard language tests including the word reading, spelling, letter-speech sound identification, letter-speech sound discrimination, rapid automized naming (RAN) and basic reaction time subtests of the 3DM battery (Dyslexia Differential Diagnosis; 3DM, [19], as well as a one-minute word reading test (EMT) [59], and reading of a short story ‘De kat’ (‘The cat’) [60]. In addition, we used a paper and pencil version of the RAVEN Coloured Progressive Matrices to assess non-verbal IQ scores (RAVEN CPM) [61]. We performed a median split of the 36 dyslexic children based on their fluency score to form groups of dysfluent dyslexic readers (n = 18) and severely dysfluent dyslexic readers (n = 18) (for similar approaches see [62]–[64]). Because our three individual word reading fluency measures were highly correlated (r >.919), reading fluency was quantified using a composite score of the 3DM word reading tests, the EMT, and “De Kat”. Subject characteristics and the results of the behavioral tests are shown in Table 1. Group differences were tested using one way ANOVAs for each of the pairwise group comparisons and are reported in the Results section.

**Table 1:** Behavioral reading scores: Descriptive data and statistical group comparisons for typical readers, dysfluent and severely dysfluent dyslexic readers.

	TypicalReaders		DysfluentReaders		SeverelyDysfluent Readers							
N	20		18		18							
Age	8.80±0.38		8.99±0.46		9.01±0.41		F(2,53) = 1.50 p = .233					
Sex ratio (m:f)	8∶12		9∶9		11∶7							
Handedness (L:R)	2∶18		3∶15		3∶15		Typical and Dysfluent		Typical and Severely Dysfluent		Dysfluent and Severely Dysfluent	
	**M**	**SD**	**M**	**SD**	**M**	**SD**	**F(1,36)** *	**p**	**F(1,36)**	**p**	**F(1,34)** *	**p**
**Word reading - ** ***accuracy*** * [%]* a												
3DM High Frequency Words - HF	99.12	1.12	95.84	3.84	90.97	6.60	13.28	**.001**	29.56	**.000**	7.31	**.011**
3DM Low Frequency Words - LF	97.25	3.23	94.58	4.30	76.33	15.31	4.72	**.036**	35.67	**.000**	23.71	**.000**
3DM Pseudowords - PW	87.37	9.65	77.27	15.26	64.80	16.07	6.07	**.019**	28.19	**.000**	5.70	**.023**
3DM Total Word Accuracy *[T]* ^ b^	49.50	9.06	39.44	9.08	25.67	8.83	11.65	**.002**	67.15	**.000**	21.29	**.000**
**Word reading – ** ***fluency***												
3DM HF*[T]*	52.95	7.58	34.44	3.45	26.94	4.28	90.16	**.000**	164.29	**.000**	33.53	**.000**
3DM LF*[T]*	54.65	9.02	35.89	2.54	26.17	4.26	72.56	**.000**	149.32	**.000**	69.15	**.000**
3 DM PW *[T]*	53.00	9.44	33.78	5.43	26.83	4.42	57.41	**.000**	115.23	**.000**	17.70	**.001**
3DM Total Word Fluency *[T]*	53.95	9.34	33.83	2.41	26.17	3.96	78.62	**.000**	136.87	**.000**	39.28	**.000**
One minute test - EMT *[C]* ^ c^	6.05	1.76	2.61	.61	1.56	.70	61.83	**.000**	102.22	**.000**	23.16	**.000**
**Story reading – ** ***fluency***												
‘De Kat’ *[T]*	54.70	8.04	36.33	2.20	29.39	5.72	87.81	**.000**	122.44	**.000**	23.12	**.000**
**Letter –speech sound coupling** *[T]*												
3DM Spelling – *accuracy*	50.60	9.14	35.89	5.96	34.22	7.45	33.66	**.000**	36.13	**.000**	.55	.464
3DM Spelling – *RT*	54.55	8.70	39.11	6.99	38.22	8.17	35.83	**.000**	35.35	**.000**	.12	.728
L-SS identification - *accuracy*	46.95	7.70	42.94	11.63	40.11	12.24	1.60	.214	4.34	**.044**	.51	.481
L-SS discrimination - *accuracy*	50.20	9.25	46.06	8.96	41.00	8.39	1.89	.177	10.23	**.003**	2.98	.094
L-SS identification – *RT*	52.80	7.08	45.00	7.39	42.56	8.08	11.03	**.002**	17.35	**.000**	.90	.350
L-SS discrimination - *RT*	51.10	8.01	46.76	9.71	49.28	9.05	2.22	.145	.43	.514	.63	.434
**Phoneme deletion** – *accuracy [T]*	52.70	7.63	41.94	7.18	36.89	8.04	19.28	**.000**	38.69	**.000**	3.83	.059
**Rapid naming (RAN)** *[T]*												
Letters	50.05	7.13	41.35	6.53	34.28	7.34	14.77	**.000**	45.12	**.000**	9.04	**.005**
Digits	50.65	10.92	40.94	6.04	35.11	8.89	10.64	**.002**	22.81	**.000**	5.09	**.031**
Pictures	49.95	7.27	40.41	7.68	41.11	12.53	15.01	**.000**	7.26	**.011**	.04	.845
RAN Total	49.85	9.91	38.76	6.86	32.83	9.15	20.36	**.000**	37.82	**.000**	4.67	**.038**
**Basic reaction time** *[T]*	60.70	9.61	62.29	7.70	66.95	10.69	.302	.586	3.59	.066	2.16	.151
**Raven matrices** *[C]*	7.04	1.49	6.81	1.68	7.34	1.25	.21	.651	.46	.503	1.19	.282

a Raw scores b T scores (M = 50, SD = 10) c C scores (M = 5, SD = 2).

* For RAN, basic reaction time, phoneme deletion and L-SS discrimination degrees of freedom were: F(1,35) and F(1,33) as data for one subject was missing.

### Stimuli

The stimuli were the same as in Froyen et al. [16], [21], [26] including auditory stimuli consisting of the Dutch vowels/a/and/o/spoken by a native Dutch female speaker. The vowels were digitally recorded (sampling rate 44.1 kHz, 16 bit quantization), band-pass filtered (180–10.000 Hz), resampled at 22.05 kHz and matched for loudness with Praat software [65]. Phoneme duration was 384 ms for vowel/a/and 348 ms for vowel/o/. Vowels were presented binaurally through headphones at a comfortable listening level of ∼65dB as measured with an analog loudness meter. The visual stimulus was a white, lower case letter “a”, presented in size 40 “Arial” font in the center of a computer screen with a black background. The stimuli were presented using Presentation 14.4 (Neurobehavioral Systems, Inc., Albany, CA).

### Experimental Design

In one auditory and two audiovisual oddball paradigms, we presented the vowel/a/as the standard (83%) and the vowel/o/as the deviant (17%) stimulus. In the auditory condition participants listened to the vowels while watching silent movies. In the two audiovisual conditions, the presentation of the standard or deviant vowel was paired with a white letter “a”, presented for 500 ms at the center of a black computer screen. Between the successive letter presentations a white fixation cross appeared in the same location. Both auditory and audiovisual trials had a trial length of 1700 ms. The two audiovisual conditions differed in the stimulus onset asynchrony (SOA) between letter and vowel presentation. In the first audiovisual condition (AV0), the letter and vowel appeared simultaneously, and in the second audiovisual condition (AV200), the letter appeared 200 ms before the vowel. During the audiovisual conditions, participants performed a simple visual target detection task to ensure fixation on the screen. The visual target consisted of a color picture of a wrapped present, which was presented instead of the letter in 3,5% of the trials (10 trials per block) and required a button press. This paradigm was the same as the one used in Froyen et al., [16], [21], [26] except for the following two changes. First, the trial length was increased from 1250 ms to 1700 ms because the experiment was part of a larger study involving coherence analysis at lower frequencies. Second, in order to obtain sufficient deviant trials within 76 minutes of measurement time, the number of deviants was increased from 10% to 17%, a percentage that was previously shown to lead to reliable mismatch responses [29], [66]. Participants performed three blocks of each condition, each block consisting of 288 trials. Per condition a total of 150 deviant and 714 standard trials were presented. Standards and deviants were presented in pseudo-random order: two successive deviants were required to be separated by at least three standards, and deviants could not be one of the first two trials of a block. The blocks of the same condition were presented in consecutive fashion, the order of the conditions was pseudorandomized and counterbalanced, with one of the cross-modal conditions always being presented first.

### EEG data recording and analysis

EEG data were recorded at a 1024 Hz sampling rate and a DC-104 Hz recording bandwidth using the Biosemi Active Two system (Biosemi, Amsterdam, Netherlands). EEG was measured from 64 active-channels, placed according to the 10–20 international system (Electro-cap International Inc., with CMS and DRL electrodes at the approximate locations of PO1 and PO2), and the CMS was used as a recording reference. Eye-movements were measured using 4 additional Flat-Type Active electrodes, 2 were placed below and above left eye and 2 at the outer canthi of each eye. In addition 2 electrodes were placed on the right and left mastoids and used for offline re-referencing. The offset range of the electrodes was kept between −20 mV and 20 mV.

For the analysis we discarded the first 2 trials of each block, as well as the first epoch after each deviant and, in the audiovisual conditions, the first 2 epochs after catch trials to account for possible movement artifacts due to button presses [67]. Per participant, we included data of the 150 deviant trials and of 150 standard trials chosen randomly from the pool of 480 standard trials. Data were analyzed using the v11.0.0b EEGLAB toolbox [68](http://www.sccn.ucsd.edu/eeglab) and custom Matlab scripts (MATLAB 2011b, The MathWorks, Natick, MA). EEG data pre-processing included offline rereferencing to the average of the left and right mastoids, applying a bandpass filter of 1–70 Hz and downsampling to 256 Hz. The EEG data was epoched from −500 ms to 1200 ms with respect to the auditory (Auditory and AV0 condition) or visual (AV200) stimulus and baseline corrected with respect to the mean signal in the 500 ms baseline period. Removal of artifacts was performed in two steps. First, the data were visually inspected and epochs containing non-stereotypical artifacts including high amplitude, high-frequency muscle noise, swallowing and electrode cable movements, were rejected. Secondly, stereotypical artifacts, including eye movements, eye blinks and heart beat artifacts were corrected with extended INFOMAX ICA [69] as implemented in EEGLAB. ICA was performed on 64 scalp channels resulting in 64 components per condition per participant which were classified as EEG or artificial activity based on scalp topography, time course and power-frequency spectra [70]. Single electrodes containing high amplitude noise were interpolated using spherical interpolation after the data was reconstructed. Component activations representing non-brain artifacts were removed, and EEG data were reconstructed from the remaining component activations representing brain activity. In typical readers, the reconstructed data was based on a mean (SD) number of 41 (4) components per subject in the auditory condition, 47 (4) in the Av0 condition and 44 (5) in the Av200 condition. The number of EEG components in the two subgroups of dyslexic children corresponded to - dysfluent dyslexics: 43 (4) components per subject in the auditory condition, 48 (4) in the Av0 condition and 46 (4) in the Av200 condition; severely dysfluent dyslexics: 42 (8) components per subject in the auditory condition, 45 (7) in the Av0 condition and 44 (7) in the Av200 condition. The reconstructed data was baseline corrected and low pass filtered at 30 Hz.

Event related potentials (ERPs) were obtained by averaging the epochs per stimulus per condition for each participant separately. In typical readers, the mean (SD) number of epochs included in the averages for the standards and deviants corresponded to 149 (1) and 149 (1) in the auditory condition, 148 (2) and 149 (2) in the AV0 condition and 148 (2) and 149 (1) in the AV200 condition. In dysfluent dyslexics these values were 149 (1) and 148 (2) in the auditory condition, 148 (2) and 148 (2) in the AV0 condition and 148 (2) and 148 (2) in the AV200 condition. Lastly, in severely dysfluent these values were 148 (5) and 148 (5) in the auditory condition, 148 (2) and 148 (2) in the AV0 condition and 148 (2) and 147 (3) in the AV200 condition.

We evaluated the presence of the mismatch negativity (MMN) and late negativity (LN) by analyzing ERPs evoked by standard and deviant stimuli in the auditory and both audiovisual conditions, in each of our subject groups. As maximal cross-modal effects were expected across fronto-central electrodes [16], [21], analysis was performed on four fronto-central sites (Fz, Cz, FC3, FC4). The timing of the MMN and LN responses was measured by determining individual peak latencies within their respective time windows: (1) 100 to 250 ms for the MMN and (2) 600 ms–750 ms for the LN [16], [21]. The amplitude of the MMN and LN responses was measured by determining the mean amplitude in the 50 ms around the individual peak latencies. We first applied mixed model ANOVAs with stimulus (2 levels: standard and deviant), condition (3 levels: Au, Av0, Av200) and electrode (4 levels) as within subjects factors and group (3 levels: typical, dysfluent dyslexic and severely dysfluent dyslexic readers) as between subjects factor. Because MMN and LN responses were detected in all conditions and all subject groups, we subsequently performed our analyses of crossmodal letter effects on the difference waves obtained by subtracting the ERPs evoked by standards from those of deviants. MMN/LN amplitude and latency measures were analyzed using mixed model ANOVAs on pairs of conditions (Au-Av0; Au-Av200) and electrode (4 levels: Fz, Cz, FC3, FC4) as within subject factors. We report Greenhouse-Geisser corrected p-values. In a further analysis, we separately analyzed the N1 and P2 responses to standard and deviant stimuli to investigate the relation between the crossmodal MMN effects and these event-related responses. N1 and P2 amplitudes were statistically assessed using repeated measures ANOVAs with Stimulus (2 levels: standard, deviant) and Electrode (4 levels: Fz, Cz, FC3, FC4) as within subject factors.

We used linear regression to investigate the relation between individual differences in ERP correlates of letter-sound associations and behavioral measures of reading fluency and related skills. ERP measures consisted of (1) MMN latency in the two audiovisual (AV0 and AV200) conditions, and (2) the MMN and LN letter-effect in the two audiovisual conditions (i.e. the MMN/LN amplitude enhancement in the audiovisual as compared to auditory conditions, [16], [21], [26]). For linear regression, ERP measures were quantified using composite scores across 4 fronto-central electrodes (Fz, Cz, FC3 and FC4). Our selection of behavioral measures of interest was based on (1) our research aim, i.e. to test the relation between reading fluency and cross-modal ERP measures, and (2) results of a large-scale study in Dutch, Hungarian and Portuguese school children in which factor analysis of test scores on 3DM subtests identified verbal working memory, phonemic awareness, RAN, letter-speech sound accuracy and letter-speech sound speed as separate factors [71]. Thus, reading fluency, was quantified using a composite score of the 3DM word reading tests, the EMT, and “De Kat”. Furthermore, the other behavioral measures included reading accuracy (3DM), letter-speech sound coupling accuracy (spelling, letter-speech sound identification and discrimination, r >.406) and speed (spelling, letter-speech sound identification and discrimination, r >.391), phonological awareness (3DM phoneme deletion accuracy) and rapid auditory naming (3DM RAN total score). We employed simple linear regressions with one of the ERP measures as a predictor and one of the behavioral composite scores as a dependent variable to probe relations of each ERP measure specifically to the behavior. As we conducted several linear regressions, i.e. 12 for latencies and 24 for amplitudes, once for all the children and once only for dyslexics, we accounted for multiple comparisons by using FDR corrections [72] and report p values relative to obtained q(FDR) thresholds.

## Results

### Behavioral tests: Typical readers, Dysfluent and Severely Dysfluent Dyslexic readers

The dyslexic readers were divided into a dysfluent and a severely dysfluent group, using a median split based on their reading fluency scores (see Methods). A comparison of behavioral test scores showed significantly lower scores for severely dysfluent as compared to dysfluent dyslexics on all word reading tests (fluency as well as accuracy), as well as on rapid automatized naming (Table 1). The dyslexic subgroups did not significantly differ on any of the other behavioral tests. Both dyslexic groups scored significantly lower than typical readers on all language tests with the exception of the accuracy of letter-speech sound discrimination and identification (comparable in dysfluent and typical readers) and the speed of letter-speech sound discrimination (comparable in all three groups). Non-verbal IQ and speed of processing (reaction times) were comparable across the three groups.

### Auditory MMN and LN effects

**Figure 1:**
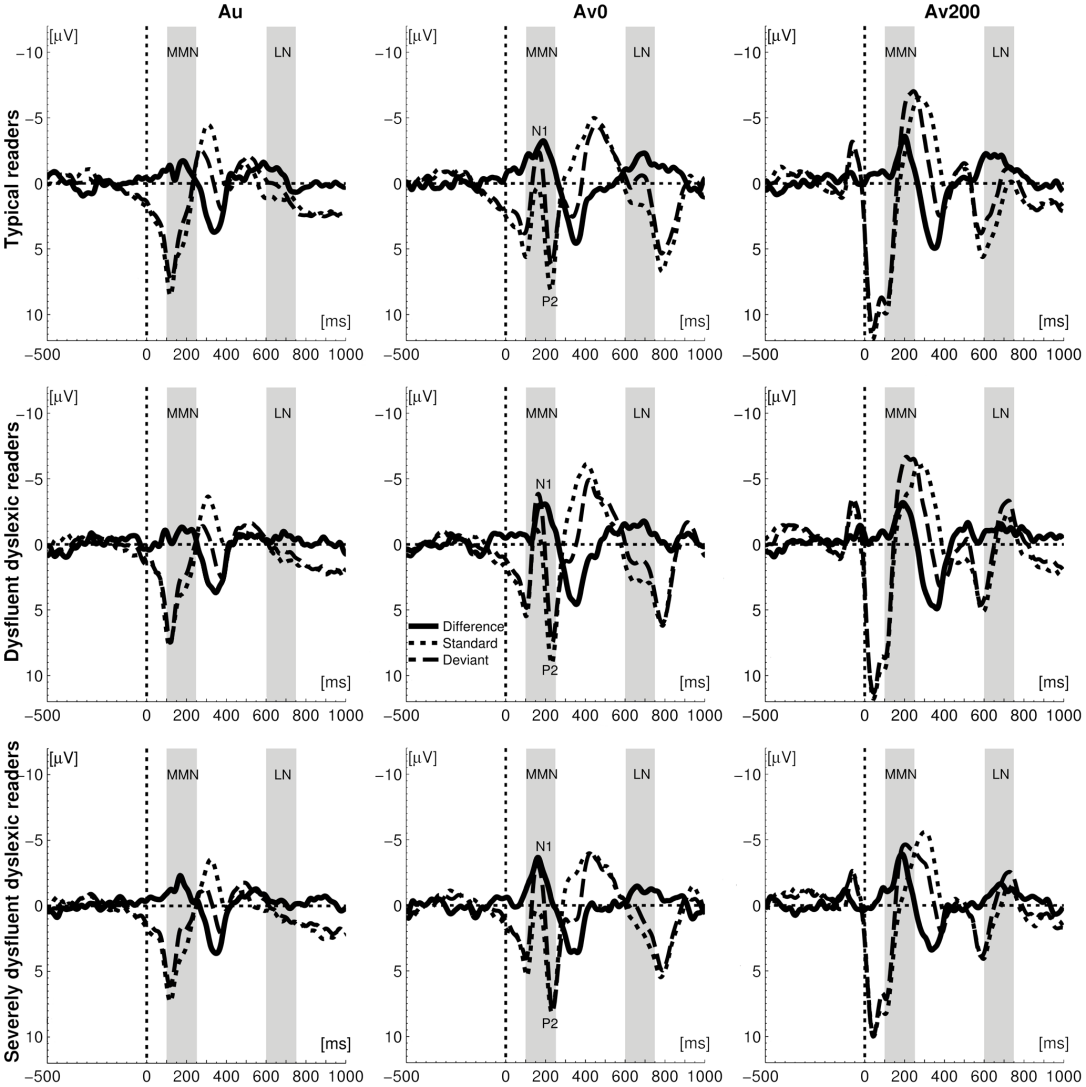
Grand average event-related potentials (ERP) and difference waves. Grand average ERPs averaged over 4 frontocentral electrodes for standard (dotted line), deviant (dashed line) and their difference (solid line) in auditory (Au) and two audiovisual conditions (Av0 and Av200) with time intervals of interest shaded in light grey. N1 and P2 peaks of standard and deviant ERPs are also marked in synchronous audiovisual condition (Av0).

**Figure 2:**
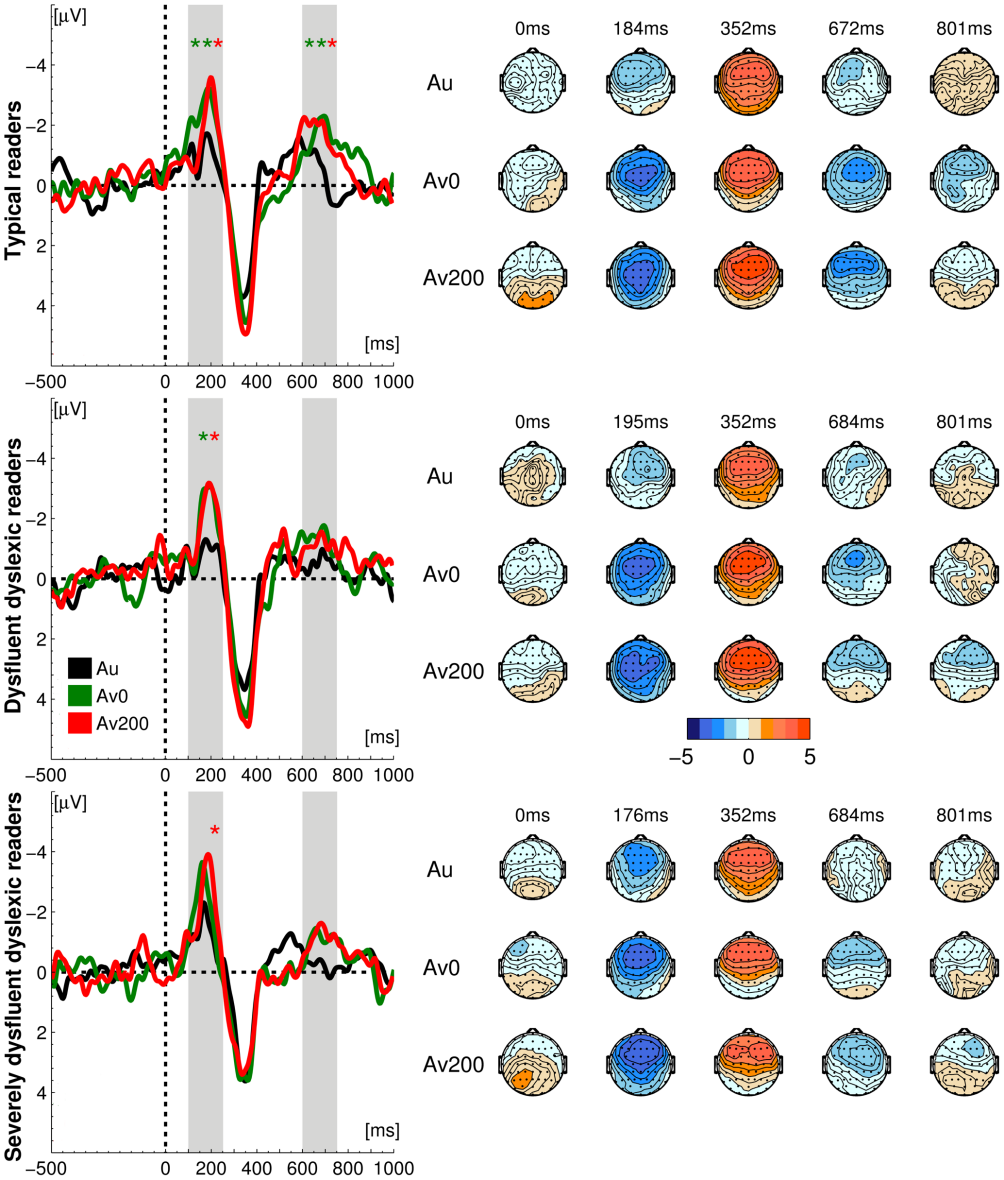
Difference waves and topographical maps. I. Difference waves (averaged over 4 frontocentral electrodes) with time intervals of interest shaded in gray. Significant differences between conditions are marked with asterisks (*p< = .05;**p< = .01): green asterisk – Av0 vs. Au difference, red asterisk – Av200 vs. Au difference. II. Topographical distribution of average amplitudes in difference waves over 64 scalp electrodes.

Because these measures served as a baseline for assessing cross-modal enhancement effects, we first analyzed the MMN and LN responses evoked in the auditory condition. In each of the three subject groups, the deviant elicited a comparable auditory MMN, with the expected fronto-central topographical distribution (Figures 1 and 2). Comparable MMN responses lead to a main effect of Stimulus (F(1, 53) = 105.26, p = .000, η^2^ = .665), without significant group differences. We also did not observe significant group differences in the latency of the auditory MMN.

In each group, the vowel deviant additionally elicited a weaker and more sustained auditory LN response around 670–690 ms after stimulus onset (Figures 1 and 2). Statistical analyses indicated a comparable auditory LN response across groups, with a significant main effect of Stimulus (F(1, 53) = 64.44, p = .000, η^2^ = .549) and no significant group differences for amplitudes or latencies. In sum, in each of the three groups the deviant vowel sound/o/elicited comparable MMN and LN responses with respect to the standard vowel sound/a/.

### Letter-effects: MMN and LN amplitude enhancements

In both cross-modal conditions (Av0 and Av200) the deviant vowel sound/o/elicited MMN and LN responses with respect to the standard vowel sound/a/(Fig. 1 and Fig. 2). In these blocks, the letter “a” was presented together with the standard and deviant vowels, leading to a double violation: the deviant speech sound/o/violated the expectation built by both the standard speech sound/a/and the letter ‘a’. To assess the neural integration of letters and speech sounds we tested whether this double violation significantly enhanced the cross-modal as compared to the auditory MMN and LN responses [16], [21], [26]. To this end, we first performed a mixed model ANOVA with stimulus (standard, deviant), condition (auditory, AV0, AV200) and electrodes (4 fronto-central electrodes) as within-subject factors and subject group as between-subject factor. In the MMN window, this overall analysis indicated stronger responses to deviants as compared to standards (Stimulus F(1,53) = 294.61, p = .000, η^2^ = .848), and stronger cross-modal as compared to auditory (mismatch) responses (Condition F(2,99) = 50.61, p = .000, η^2^ = .488; Stimulus*Condition: F(2,105) = 12.29, p = .000, η^2^ = .188), as well as a tendency for groups to differ in their response amplitudes across conditions (Condition*Group (F(4,99) = 2.18, p = .081, η^2^ = .076). Pairwise group comparisons showed that this tendency was driven by significant differences between severely dysfluent dyslexics and typical readers (Condition*Group: F(2,69) = 3.87, p = .027, η^2^ = .097), while there were no differences in other cases.

Also in the later LN window, the overall mixed-model ANOVA indicated stronger responses to deviants as compared to standards (Stimulus F(1,53) = 267.28, p = .000, η^2^ = .835) and stronger cross-modal as compared to auditory (mismatch) responses (Condition F(1,73) = 9.47, p = .001, η^2^ = .152; Stimulus*Condition: F(2,99) = 8.78, p = .000, η^2^ = .142) together with significant group differences in response amplitudes across conditions (Condition*Group: F(3,73) = 3.87, p = .015, η^2^ = .127). Pairwise group comparisons showed that the significant group difference in response amplitudes across conditions was driven by significant differences between severely dysfluent dyslexics and typical readers (Condition*Group: F(1,51) = 4.49, p = .027, η^2^ = .111) as well as between dysfluent dyslexics and typical readers (Condition*Group: F(1,49) = 6.80, p = .006, η^2^ = .159), but not by a difference between the two dyslexic groups (F(1,46) = 0.48, p = .549, η^2^ = .014).

We further analyzed these condition and group effects by performing mixed model ANOVAs on the MMN difference waves with Condition (AV0 versus Auditory; AV200 versus Auditory) and electrodes (4 fronto-central electrodes) as within subjects factors. In the simultaneous cross-modal condition (AV0), significant MMN amplitude enhancements were observed in typical readers (F(1,19) = 10.92, p = .004, η^2^ = .365) as well as in dysfluent dyslexics (F(1,17) = 7.63, p = .013, η^2^ = .310), whereas in severely dysfluent dyslexics this enhancement was not significant (F(1,17) = 3.43, p = .082, η^2^ = .168). When letter presentation preceded speech sound onset (AV200) significant cross-modal MMN enhancements were found in all three subject groups (typical readers: F(1,19) = 7.21, p = .015, η^2^ = .275; dysfluent dyslexics: F(1,17) = 5.93, p = .026, η^2^ = .259; severely dysfluent dyslexics: F(1,17) = 6.09, p = .024, η^2^ = .264).

As for the LN window, in typical readers the presence of a letter significantly enhanced the LN amplitude in both cross-modal conditions (AV0 vs. Auditory: F(1,19) = 10.90, p = .004, η^2^ = .365; AV200 vs. Auditory: F(1,19) = 7.85, p = .011, η^2^ = .292). In contrast, in the dyslexic groups the LN letter effects only resulted in non-significant trends (AV0 vs. Auditory, dysfluent: F(1,17) = 3.93, p = .064, η^2^ = .188; severely dysfluent: F(1,17) = 3.98, p = .062, η^2^ = .190; Av200 vs. Auditory, dysfluent: (F(1,17) = 3.44, p = .081, η^2^ = .168; severely dysfluent: F(1,17) = 2.41, p = .139, η^2^ = .124).

In summary, in both cross-modal conditions, our results showed early (MMN) and late (LN) letter effects in typical readers. Although the group differences in MMN and LN letter effects were subtle, the dysfluent group resembled typical readers in the presence of an MMN letter effect, whereas they resembled the severely dysfluent group in the lack of a significant LN letter effect. The severely dysfluent group differed most from typical readers in both the lack of a significant LN letter effect and in only showing a significant MMN letter effect in the AV200 condition.

### Letter effects: MMN and LN latency

We investigated the timing of the letter effects, by analyzing MMN and LN latencies in both crossmodal conditions. As already indicated by our regression analysis, groups tended to differ in MMN response latency in the simultaneous crossmodal (AV0) condition (Group (F(2,53) = 5.85, p = .062, η^2^ = .181). In particular, severely dysfluent dyslexics showed a significantly shorter MMN latency than both the typical readers (F(1,36) = 8.59, p = .006, η^2^ = .193), and the dysfluent dyslexics (F(1,34) = 7.65, p = .009, η^2^ = .184), whereas the latter groups showed similar latencies (F(1,36) = 0.07, p = .787, η^2^ = .002). No significant group differences were found for MMN latency in the asynchronous cross-modal condition (AV200), or for LN latency in either of the cross-modal conditions.

### ERP - behavior relations

**Figure 3:**
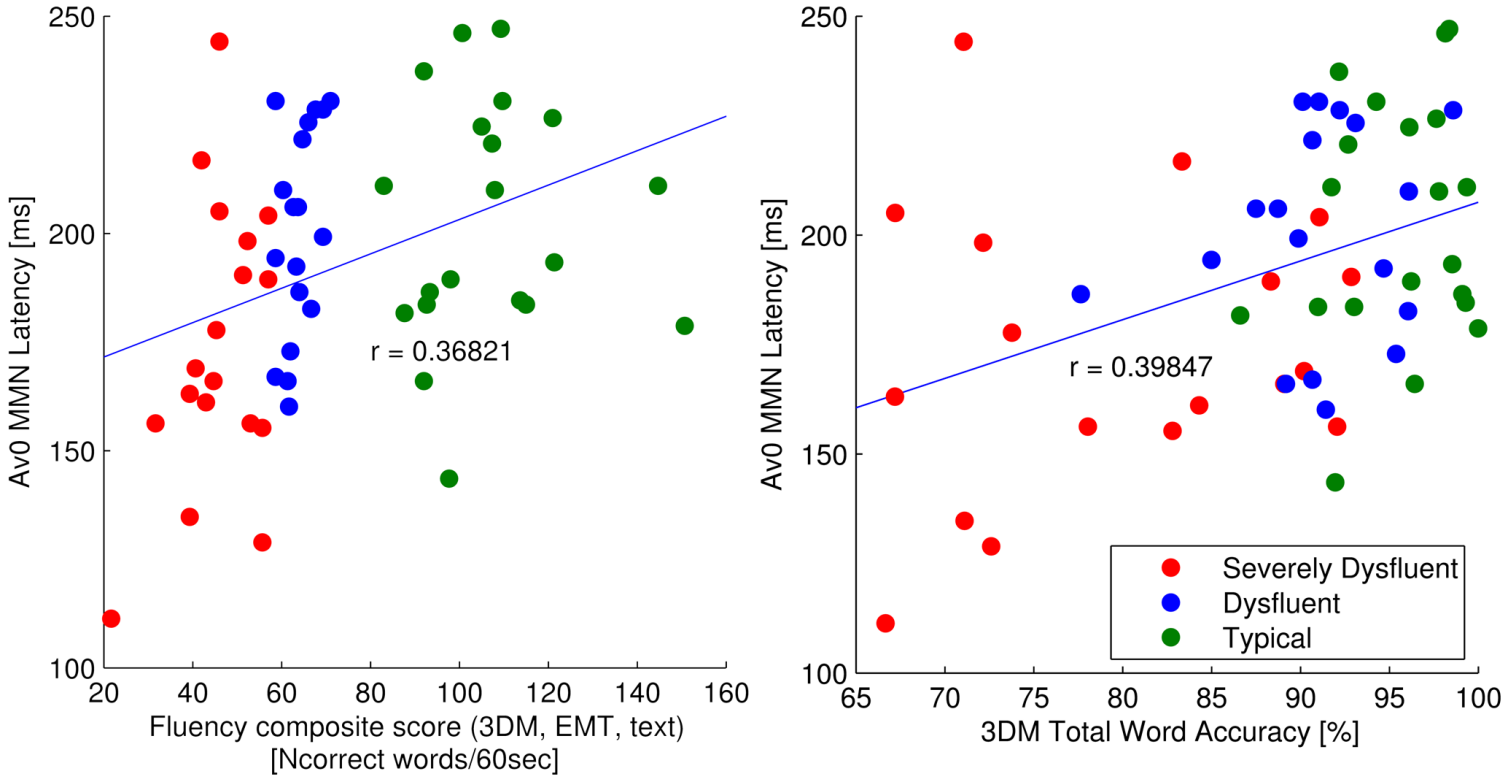
Correlations of word reading fluency and accuracy with the MMN latency in Av0 condition. Relation of composite fluency (3DM word reading fluency, EMT, ‘De Kat’) and 3DM Accuracy (HFW, LFW, PW) raw scores with MMN latency in Av0 condition with the strength of correlation represented by r.

We used linear regression to investigate the relation between individual differences in MMN/LN responses in the crossmodal conditions and behavioral measures of reading fluency and related skills. Results showed that such a relation was present for the latency of the MMN response elicited in the simultaneous crossmodal condition (AV0). First, across both typical and dyslexic readers, MMN latency in the AV0 condition significantly correlated (significance threshold q(FDR) = .010) with individual differences in word reading fluency (r = .368; p = .005; Table 2, Figure 3), word reading accuracy (r = .398; p = .002) and phoneme deletion accuracy (r = .343; p = .001). When restricting the analysis to the dyslexic readers, a significant relation was only shown for reading fluency (r = .554; p = .000). Remarkably, in both cases, longer MMN peak latencies were found in the more fluent readers, a result that is further investigated in our analysis of the N1-P2 responses underlying the MMN effect. None of the other ERP-behavior regressions reached statistical significance.

**Table 2:** Regression of word reading fluency and accuracy with the MMN latency in Av0 condition: Regression coefficients for behavioral measures and latency of MMN in Av0 condition averaged over 4 electrodes (significant p values for q(FDR) = .010 are denoted in bold).

	Av0 MMN latency *[ms]*							
	All children				Dyslexic children			
	R^2^	β	t(54)	p	R^2^	β	t(34)	p
**Word reading – ** ***fluency***								
M_3DM Total Word Fluency, EMT, ‘De Kat’_	.136	.368	2.91	**.005**	.307	.554	3.88	**.000**
**Word reading - ** ***accuracy*** * [%]*								
3DM Total Word Accuracy	.159	.398	3.19	**.002**	.139	.372	2.34	.025
**Letter –speech sound coupling**								
M_3DM_ _Spelling, L-SS identification, L-SS discrimination_ – *accuracy [%]*	.073	.271	2.05	.046	.044	.209	1.23	.227
M_3DM Spelling, L-SS identification, L-SS discrimination_ – *RT [sec/item]*	.031	−.176	−1.30	.199	.000	.006	0.03	.975
**Phoneme deletion** – *accuracy [%]*	.117	.343	2.66	**.010**	.089	.298	1.80	.082
**Rapid naming (RAN)** *[ms]*	.103	−.321	−2.47	.017	.095	−.308	−1.86	.072

### Relation between MMN and N1-P2 responses

In a final analysis we aimed to further investigate the origin of the *shorter* MMN latency in severely dysfluent dyslexics as compared to both other groups, as well as the corresponding regression results in the simultaneous cross-modal (AV0) condition (Figure 3) by analyzing the timing of the MMN with respect to the evoked N1 and P2 responses (Figure 1). Results showed that in severely dysfluent dyslexics, letter-speech sound pairs only elicited a deviancy effect in the N1 window (main effect of Stimulus F(1,17) = 28.32, p = .000, η^2^ = .625), but not in the P2 window (F(1,17) = .03, p = .860, η^2^ = .002), as can also be seen in Figure 1. In contrast, typical readers as well as dysfluent dyslexics showed a deviancy effect in both the N1 and the P2 windows (main effects of Stimulus typical readers N1: F(1,19) = 32.27, p = .000, η^2^ = .629; P2: F(1,19) = 6.49, p = .020, η^2^ = .255); dysfluent dyslexics N1: F(1,17) = 14.24, p = .002, η^2^ = .456 and P2: F(1,17) = 10.15, p = .005, η^2^ = .374). The absence of a P2 deviancy effect in the severely dysfluent dyslexics thus most likely explains their shorter MMN latency.

## Discussion

The present study investigated ERP measures of letter-speech sound integration in typically reading and dyslexic children after 2,5 years of reading instruction and specifically investigated how these ERP measures relate to individual differences in reading fluency and related skills. For this purpose we employed a passive oddball paradigm and tested cross-modal enhancement of the MMN and LN due to an audiovisual violation (vowel sound/o/vs. vowel sound/a/and letter ‘a’) as compared to an auditory only violation (vowel sound/o/vs. vowel sound/a/; [21]). Our results show robust neural integration of letters and speech sounds in the typical readers, albeit at a different temporal integration window compared to adults [26]. Furthermore, they confirm and extend previous findings in dyslexic children by demonstrating reduced letter-speech sound integration, with different patterns of integration deficiency depending on the level of reading dysfluency (Table 3).

**Table 3:** Summary of results.

	Severely dysfluent dyslexic	Dysfluent dyslexic	Typical
**MMN**			
Av0 vs. Au	**No**	Yes	Yes
Av200 vs. Au	Yes	Yes	Yes
**LN**			
Av0 vs. Au	**No**	**No**	Yes
Av200 vs. Au	**No**	**No**	Yes
**Av0**			
N1 difference	Yes	Yes	Yes
P2 difference	**No**	Yes	Yes

### Typical readers: enhanced (and non-selective) neural sensitivity?

Typical readers after 2,5 years of reading instruction showed cross-modal enhancements of the MMN and LN responses independent of the precise timing of letter and speech sound presentation (AV0 and Av200), indicating a broad temporal window of integration. This contrasts with previous results of adults, who only showed a cross-modal enhancement in the MMN window, and only when letters and speech sounds were presented simultaneously [26]. Furthermore, the present results showed a different pattern of cross-modal effects as compared to previous findings in both typical beginning readers after 1-year of reading instruction and advanced typical readers after 4-years of reading instruction [21]. That is, in the younger readers cross-modal enhancement only reached significance in the LN window and only when the letter preceded the speech sound (AV200). In the advanced readers this pattern shifted with significant cross-modal enhancement in the earlier MMN window when the letter preceded the speech sound (AV200) and additionally in the LN window when letters and speech sounds were presented simultaneously (AV0). In comparison to the younger readers, the observed emergence of a cross-modal MMN enhancement may reflect the initial development of an early detection and integration of letter-sound pairs in 9-year-old children [27], [28]. In comparison to the more advanced readers, who only showed a cross-modal LN enhancement in the simultaneous AV0 condition, the unspecific cross-modal LN enhancement independent of the timing of letter presentation, may suggest immature and non-selective further processing of letter-sound congruency in 9-year-olds. On a more general level, the pattern of stronger and/or less selective cross-modal effects in our intermediate group of typical readers is consistent with a developing neural system for audiovisual speech and letter-sound processing that undergoes dynamic changes throughout primary school years [6], [73], [74]. In particular, they may reflect enhanced neural sensitivity for letter-sound congruency after 2.5 years of reading instruction, before developing into a more selectively tuned system with advanced reading practice. Similar nonlinear patterns of an initial sensitivity increase during early school years, followed by reduced and more selective sensitivity in adults, have been observed for print selective visual N1 responses [53] and ERP phonological priming effects during an auditory lexical decision task [52]. On the other hand, also methodological differences between the present study and the previous ERP studies by Froyen and colleagues [16], [21], [26] may have (at least partly) contributed to the relatively strong cross-modal ERP effects in our typical readers. For example, unlike these previous studies, we used active electrodes for EEG data acquisition and ICA-based EEG preprocessing, which both lead to improvement of ERP signal-to-noise ratio. Another factor that may have increased signal-to-noise ratio is an increase in trial length from 1250 ms to 1700 ms. In addition, we employed a full within-subjects design as compared to a partial within-subjects design [21]. To further understand the development of brain mechanisms for letter-speech sound integration and their relation to reading acquisition, it is important to apply similar cross-modal neuroimaging paradigms in a longitudinal set-up and follow the same children while they are learning to read.

### Dyslexic children: crossmodal MMN effect scales with reading dysfluency

ERP data of dyslexic children showed normal auditory MMN and LN responses together with reduced effects of letter-speech sound congruency. Most interestingly results indicated different patterns of integration deficiency depending on the level of reading fluency. In particular, in our regression analysis behavioral measures of reading fluency significantly predicted the latency of the MMN response in the simultaneous cross-modal condition (AV0), not only across typical and dyslexic readers, but also within the group of dyslexic readers. Further analyses indicated that this relation could be explained by a short-lasting, reduced MMN response, encompassing only the N1 window in severely dysfluent dyslexics as compared to a longer lasting MMN response, encompassing both the N1 and P2 windows in dysfluent dyslexic and typical readers. In addition, in the AV0 condition, MMN latency correlated significantly with reading accuracy and phonological awareness (phoneme deletion) across typical readers and dyslexics, but not within the dyslexic group alone.

Although relatively little is known about the functional relevance of the P2, recent evidence suggests that this response is specifically sensitive to the audiovisual integration of orthographic and phonological units [47] as well as of visual articulatory gestures and speech [74]–[77]. For example, in an elegant study by Baart et al. [75], audiovisual integration of articulatory gestures (lipread speech) and sine-wave speech was compared between participants trained to perceive the sine-wave speech in either a ‘speech’ or ‘nonspeech’ mode. Whereas lipread speech modulated the N1 response in both processing modes, the P2 was only modulated in listeners who recognized the sine-wave speech as speech, thus suggesting more general audiovisual convergence in the N1 window followed by speech specific audiovisual integration in the P2 window. This speech specificity of the P2 response would also concur with its putative neural source in the posterior superior temporal sulcus [78], a region involved in the integration of audiovisual speech [79] and of letters and speech sounds [23], [80]. Moreover activity in this region has been found to scale with interindividual differences in audiovisual speech perception (McGurk effect) in typically reading children and adults [73], [81] and to show reduced letter-speech sound integration in dyslexic children [15]. By analogy, we would speculate that the N1 deviancy effect shown in the simultaneous audiovisual (AV0) condition indicates audiovisual convergence common across the three children groups, followed by a speech specific integration of letters and speech sounds in the P2 window in typical readers and dysfluent dyslexics, which is reduced or absent in severely dysfluent dyslexics. Recently, a diminished speech-specificity of audiovisual integration was also found for spoken-written syllable pairs in Finnish dyslexic adults [41].

A similar pattern of results or relation with behavior (reading fluency) was not observed in the asynchronous crossmodal (AV200) blocks, possibly indicating a diminished recruitment of speech specific integration processes in this condition. This may also explain why typical adult readers, with selectively tuned neural systems for letter-speech sound integration, only show congruency effects when letters and speech sounds are presented simultaneously (AV0 blocks, [23], [26]). These findings deserve further study however, especially in relation to the nature of possible audiovisual integration deficits in dyslexics, because in a previous study with 11 year old dyslexic readers, behavioral measures of word reading fluency (MMN), non-word reading fluency and letter-phoneme matching (LN) were found to correlate with MMN/LN letter effects in the AV200 condition [16].

Further evidence for brain-behavior relations with respect to letter-speech sound integration comes mainly from fMRI studies. [15], for example found a positive correlation between letter-speech sound congruency effects measured in the planum temporale/Heschl’s sulcus and both reading accuracy and the speed of letter-speech sound matching across 9-year old typical and dyslexic readers. Cross-modal fMRI congruency effects measured in the planum temporale during a rhyme judgment task were also found to positively correlate with literacy skills [82] as well as with phonological awareness [17] in 8–11 year old typical readers. However, in the latter study no correlation was found between cross-modal integration and phonological awareness in dyslexic children, which was suggested to indicate a decoupling of these processes in dyslexia [17]. Although most studies thus find a relation between reading and/or phonological skills and ERP/fMRI indices of letter-speech sound integration, the type of relation shows some variability as well as its presence or absence in dyslexic readers. These differences may relate to the dependency of neural effects of letter-speech sound congruency on task demands [25], [46] the type of speech/letter units, e.g. consonants, vowels, syllables [38], [83] and/or the depth of the orthography [84]. Importantly, the present results show that the severity of reading (dys)fluency may also lead to differences in the observed brain-behavior correlations across studies. It would thus be interesting to further investigate these brain-behavior relationships in a larger-scale cross-national study including children of varying reading (dys)fluency, and deep and shallow orthographies, using the same paradigm with stimuli of grain sizes with different relevance in the different orthographies. Furthermore, the observed relation between reading fluency/accuracy and crossmodal ERP modulations across typical as well as dyslexic readers is compatible with a continuity model of reading dysfunction [80], although our results do not exclude the possibility of dyslexia subtypes.

Given the present focus on letter-speech sound integration, it may seem surprising that behavioral measures of letter-speech sound coupling showed the weakest differences across reading groups and did not correlate with ERP measures of letter-speech sound coupling. However, these findings are in agreement with previous ERP and fMRI evidence revealing a dissociation between children’s knowledge of which letters belong to which speech sounds and the automatic neural integration of these associations [19]–[21]. The present results thus provide additional support for the notion that behavioral measures alone may not to be a sensitive indicator for letter–speech sound integration [6], [21].

### Normal auditory responses and subtle reduction of LN letter effect in dyslexics

Results showed comparable auditory MMN and LN responses to vowels in all three groups. This absence of group differences was expected because vowels typically elicit normal MMN and/or LN responses in dyslexic children [16], [85] whereas deficient change detection responses are typically reported for more subtle speech changes involving e.g. stop consonants (e.g. [85], [86]). Correspondingly, we did not find any correlations between auditory MMN and LN responses and behavioral measures of reading or reading-related skills.

Our results also confirm weak neural integration of letters and speech sounds in 9 year old dyslexic children [15], with reduced or absent audiovisual LN effects in both groups and a short-lived MMN effect in the AV0 condition in severely dysfluent dyslexics. The timing of these effects was different than those previously reported in 11 year old dyslexics, for whom MMN letter effects did not, but the LN letter effect in the AV200 condition did, reach significance [16]. These differences could relate to developmental or strategy changes in dyslexic readers after 2.5 versus 4 years of reading instruction, but could also relate to methodological differences between the two studies and/or differences in the severity of reading dysfluency (see above).

Interestingly, an attenuated late negativity to spoken/ba/deviants as compared to/da/standards has been proposed as a potential endophenotype for dyslexia [36]. That is, this negativity in a broader time window (300–700 ms) overlapping with our crossmodal LN (600–750 ms) was found to be attenuated in both dyslexic children and their siblings without dyslexia [36], and to be associated with rare variants in a candidate gene region for dyslexia [43]. As compared to perceptual aspects of letter-speech sound congruency in the MMN window, the observed LN letter effect may reflect cognitive, explicit associative and/or attentional processes [16], [21], [36], [87], present depending on familiarity and complexity of the stimuli. Whereas in adults these type of late orthographic-phonological interactions may only occur during complex metaphonological tasks [46], [47] and pseudoword-word priming tasks [51], in typically reading children they seem to be recruited during the integration of simple letter-vowel pairs (present findings and [21]), letter strings [88], integration of audiovisual words [89] and a visual lexical decision task with phonological distractors [49], with disrupted recruitment in dyslexic children (present findings and [16], [49]). If this process is disrupted, as was the case in both dyslexic groups then the automaticity in adulthood may not be reached [18], as it may be prevented by an incapability to access and/or manipulate the representations [15], [51], [90], and/or reduced attentionally-mediated integration [36], [43], [50], [51]. The additional attenuation of the early MMN in severely dyslexic children may signal a more basic failure in forming a clear letter-speech sound representation [6], and concurs with the suggested timing of sublexical orthographic-phonological integration during online visual word recognition [91]. Similarly, Roeske and colleagues [92] observed an association between specific genetic markers and a speech evoked late negativity across dyslexic children independently of the severity of their reading problems, while a significant association with the earlier speech evoked MMN was present only in the most severe dyslexics. Thus, the crossmodal MMN enhancement would represent a successful representation of an audiovisual stimulus, and the crossmodal LN enhancement would characterize availability of the represented stimulus for further manipulation.

Finally, Blau and colleagues [15] showed that Dutch dyslexic children of the same age as our participants, exhibit reduced neural integration of letters and speech sounds in the planum temporale/Heschl sulcus and the superior temporal sulcus, thus showing both reduced integration in the STS and feedback to the auditory cortex [23]. As the severely dysfluent dyslexics in our study showed both early and late deficiencies, while dysfluent dyslexics showed only a late deficiency, it would be interesting to investigate whether the early MMN deficiency would stem from the reduced integration in the STS while the LN deficiency would be a product of a reduced feedback.

## Conclusion

The present ERP study investigated letter-speech sound integration in 9-year-old typically reading and dyslexic children by using a well-studied oddball paradigm with simple vowel sounds and letters and demonstrated that early (speech specific) audiovisual integration processes scale with individual differences in reading (dys)fluency. Results further indicated enhanced neural sensitivity to letter-speech sound associations in 9-year-old typical readers, together with disrupted sensitivity in dyslexic readers. In future studies, this ERP paradigm could be used to investigate whether and how systematic training of phonological skills and/or letter speech-sound coupling changes this disrupted neural integration of letters and speech sounds in dyslexic children. Furthermore, an extension to longitudinal designs using units of different complexity and grain sizes (e.g. vowels, consonants, syllables, words), possibly in the context of more naturalistic on-line reading tasks, could lead to a more complete understanding of the dynamic neural changes contributing to successful and hampered reading acquisition.
